# Involvement of Netrins and Their Receptors in Neuronal Migration in the Cerebral Cortex

**DOI:** 10.3389/fcell.2020.590009

**Published:** 2021-01-15

**Authors:** Satoru Yamagishi, Yuki Bando, Kohji Sato

**Affiliations:** Department of Organ and Tissue Anatomy, Hamamatsu University School of Medicine, Hamamatsu, Japan

**Keywords:** axon guidance, netrin, DCC, Unc5, neogenin

## Abstract

In mammals, excitatory cortical neurons develop from the proliferative epithelium and progenitor cells in the ventricular zone and subventricular zone, and migrate radially to the cortical plate, whereas inhibitory GABAergic interneurons are born in the ganglionic eminence and migrate tangentially. The migration of newly born cortical neurons is tightly regulated by both extracellular and intracellular signaling to ensure proper positioning and projections. Non-cell-autonomous extracellular molecules, such as growth factors, axon guidance molecules, extracellular matrix, and other ligands, play a role in cortical migration, either by acting as attractants or repellents. In this article, we review the guidance molecules that act as cell–cell recognition molecules for the regulation of neuronal migration, with a focus on netrin family proteins, their receptors, and related molecules, including neogenin, repulsive guidance molecules (RGMs), Down syndrome cell adhesion molecule (DSCAM), fibronectin leucine-rich repeat transmembrane proteins (FLRTs), and draxin. Netrin proteins induce attractive and repulsive signals depending on their receptors. For example, binding of netrin-1 to deleted in colorectal cancer (DCC), possibly together with Unc5, repels migrating GABAergic neurons from the ventricular zone of the ganglionic eminence, whereas binding to α3β1 integrin promotes cortical interneuron migration. Human genetic disorders associated with these and related guidance molecules, such as congenital mirror movements, schizophrenia, and bipolar disorder, are also discussed.

## Cortical Neuron Migration

The mammalian cerebral cortex is a highly organized laminar structure with six layers, each of which contains a characteristic distribution of different neurons with various connections to other cortical and subcortical regions. During development, excitatory neurons are generated from radial glia and progenitor cells in the ventricular zone (VZ) and subventricular zone (SVZ), and migrate radially toward the cortical plate in an inside-out pattern (Lui et al., 2011), whereas inhibitory GABAergic interneurons are born in the ganglionic eminence and migrate tangentially (Xu et al., 2004). Distinct subtypes of cortical GABAergic interneurons are generated in specific regions of the basal telencephalon. Parvalbumin- and somatostatin-expressing interneurons derive from the lateral and medial ganglionic eminence, while most calretinin-positive interneurons are born at later stages in the caudal ganglionic eminence (Xu et al., 2004; Butt et al., 2005; Miyoshi and Fishell, 2011).

Radial glia can be classified as apical radial glia, which connect to the apical surface (lateral ventricle) with short processes and to the basal side (outer surface) with long processes, and basal radial glia, which have no apical processes and are located in the SVZ. Radial glia undergo asymmetric division, generating a progenitor cell or excitatory neuron. Intermediate progenitors are derived from radial glia disconnected from the ventricular surface and generate neurons after undergoing multiple rounds of symmetric cell division (Miyata et al., 2001; Haubensak et al., 2004; Noctor et al., 2004; Lui et al., 2011). Newly generated neurons have multipolar processes and migrate relatively slowly in the intermediate zone but transition to a bipolar morphology once they enter the cortical plate, where they migrate radially and rapidly along the basal processes of radial glia in an inside-out manner. Therefore, early born neurons (∼embryonic day 12 [E12] in rodents) form the deeper layers, and later born neurons migrate to the upper layers, stopping just below layer I by Reelin signaling (Hirota and Nakajima, 2017).

The migration of excitatory and inhibitory neurons is precisely organized by extracellular cues, including guidance molecules such as netrins, ephrins, semaphorins, and slits. These molecules are well-known for navigating axonal growth cones, but they can also regulate cell migration using similar ligand-receptor binding systems. In this review, we focus on netrin family proteins, their receptors, and related molecules, and describe the mechanisms by which migrating neurons in mammalian cerebral cortex utilize those molecules to navigate to their final destinations. Finally, we discuss the human genetic disorders of these guidance molecules, such as congenital mirror movements, schizophrenia, and bipolar disorder.

## Netrin Family Proteins

**FIGURE 1:**
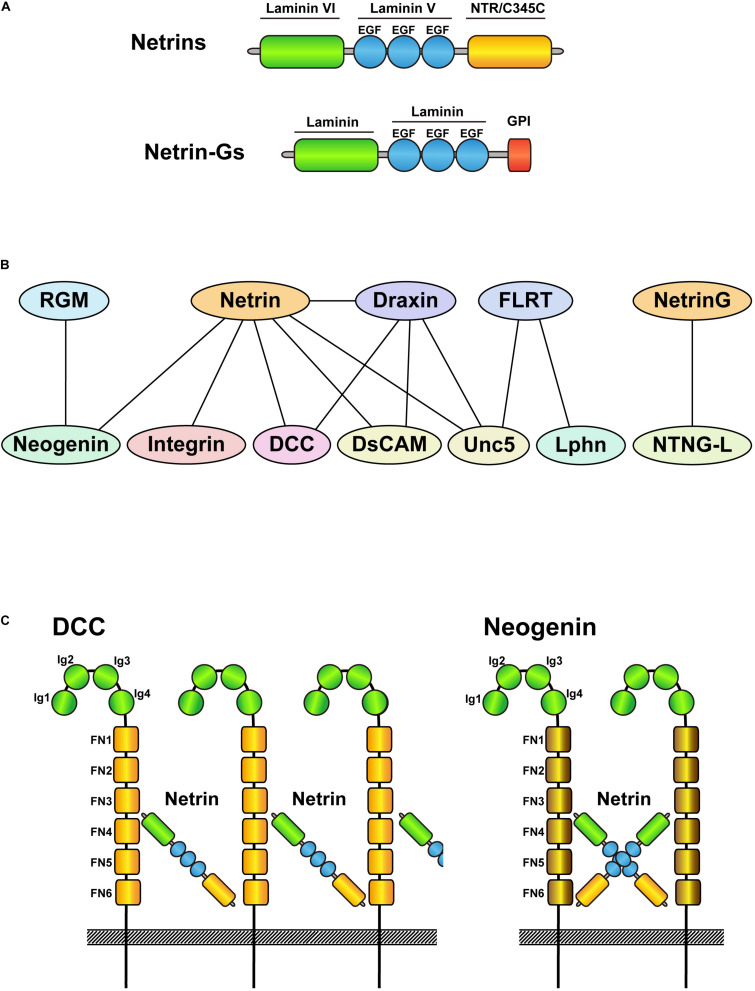
Schematic drawings of netrin family proteins and their interacting proteins. **(A)** Schematic drawing of the domain structure of netrins and netrin-Gs. Netrins are secreted proteins, whereas netrin-Gs are membrane bound GPI-anchored proteins. **(B)** Protein interactions of netrin family proteins and their receptors. Upper side indicates ligands and lower side shows their receptors. Note that netrin-Gs bind to netrin-G ligands as receptors. **(C)** Schematic drawings of the interactions between netrin-DCC and netrin-neogenin. Although DCC and neogenin are structurally similar, netrin-DCC binds continuously and makes a large complex, whereas netrin-neogenin forms a 2:2 complex.

Netrin was first identified as Unc-6 (uncoordinated), which regulates neural development in *Caenorhabditis elegans* (Ishii et al., 1992). It was named from the Sanskrit word *netr*, meaning “the person who guides.” It is conserved among non-vertebrate and vertebrate species (Lai Wing Sun et al., 2011), including mammals, which express netrin family members netrin-1, -3, -4, -5, G1, and G2. Netrin-2 expression has only been identified in chickens and fish (Kennedy et al., 1994; Serafini et al., 1994; Park et al., 2005). Netrin-1, -3, -4, and -5 are secreted, whereas netrin G1 and G2 have a glycophosphatidylinositol anchor region that binds them to the membrane. Netrins typically contain an N-terminal laminin VI domain, laminin V-type EGF-like domains, and the NTR/C345C domain (Figure 1A). However, netrin-5 lacks portions of those domains depending on the splicing variant (Visser et al., 2015; Yamagishi et al., 2015). Netrin-1 was considered a typical guidance cue that attracts axons of commissural neurons growing dorsoventrally from the roof plate toward the floor plate in the spinal cord, where it is highly expressed. However, two independent groups recently showed that netrin-1 expression at the floor plate is dispensable for this wiring (Dominici et al., 2017; Varadarajan et al., 2017). Using conditional knockout mice, they showed that netrin-1 supplied from the VZ is required for growth of commissural axons, functioning as a local cue but not as a long-range attractant.

Netrin-1 is an important attractant and repellant for axon guidance depending on its receptors (DCC, neogenin, Unc5, DSCAM, and integrins; Figure 1B). DCC and neogenin both contain four Ig-like C2 domains and six fibronectin type-III domains, with 50% amino acid homology. However, their crystal structures revealed different architectures when bound to netrin-1 (Figure 1C). Whereas the netrin/DCC complex is constructed as a continuous netrin-DCC-netrin-DCC-repeating assembly, netrin/neogenin forms a 2:2 heterotetramer complex (Xu et al., 2014). Furthermore, neogenin is also a receptor for the repulsive guidance molecule a/b (RGM a/b), which does not bind DCC. Another well-known repulsive binding interaction, netrin/Unc5, has been characterized by crystallography (Grandin et al., 2016). The V2 domain of netrin-1 binds to the Ig1/Ig2 domain of Unc5B, which can bind and compete with FLRT proteins (other Unc5 ligands; Figure 1B) (Shirakawa et al., 2019). The different patterns of binding to receptors likely contribute to the variety of netrin functions, such as cell migration, axon branching, synaptogenesis, oligodendrocyte differentiation, angiogenesis, lymphangiogenesis, immune function, and tumor progression (Rajasekharan and Kennedy, 2009; Larrieu-Lahargue et al., 2011; Finci et al., 2015; Feinstein and Ramkhelawon, 2017; Bruikman et al., 2019; Meijers et al., 2020).

## Roles of Netrin Family Proteins in the Migration of Cortical Neurons

**FIGURE 2:**
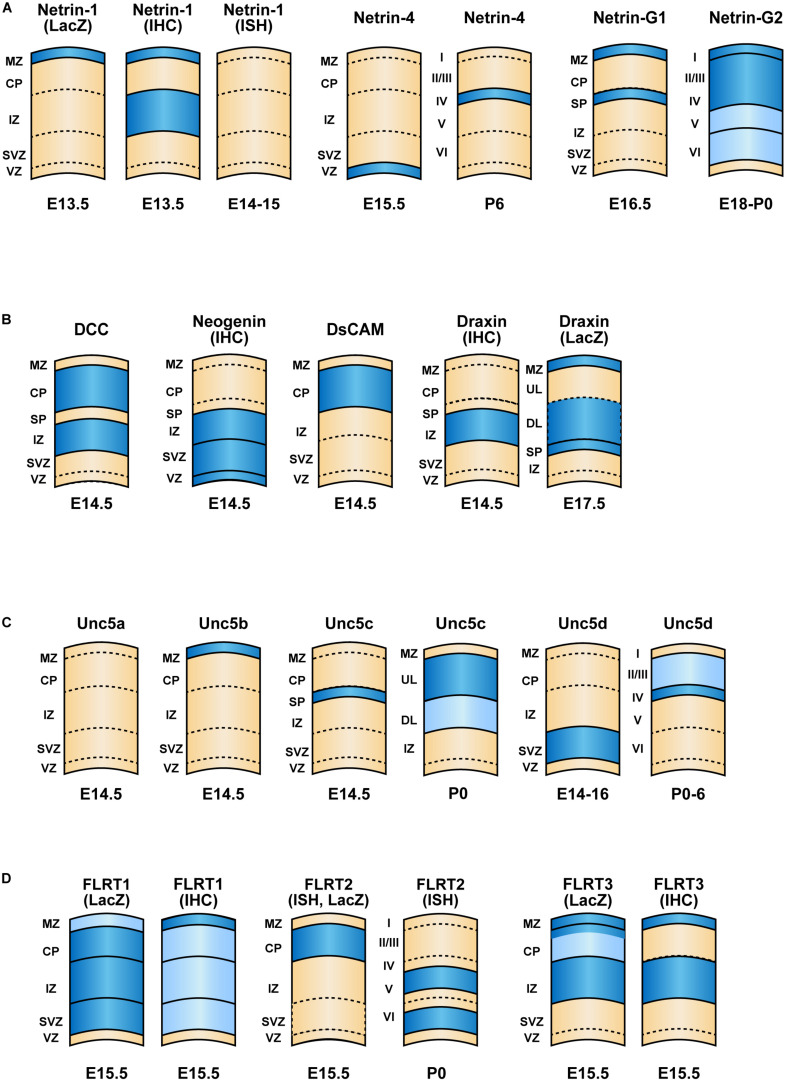
Expression patterns of netrin family proteins and their interacting proteins in the cerebral cortex. Schematic of cortical layers depicting the laminar-specific expression of **(A)** netrins, **(B)** their interacting proteins (DCC, neogenin, DsCAM and Draxin, **(C)** Unc5 family receptors and **(D)** FLRT family proteins within the neocortex. Dark blue and light blue indicate higher and lower relative levels of expression, respectively. The expression levels are based on results of *in situ* hybridization unless mentioned otherwise. The development stages are indicated. References to publications on each of the genes are listed in Table 1. ISH, *in situ* hybridization; IHC, immunohistochemistry.

Netrin-1 is involved in the migration of GABAergic interneurons. In the developing mouse, netrin-1 is highly expressed in the VZ of the ganglionic eminence and expressed at a lower level in the marginal zone and intermediate zone of the cerebral cortex at the mid- to late-gestational stage (Hamasaki et al., 2001; Stanco et al., 2009). Hamasaki et al. (2001) showed that netrin-1 repels postmitotic GABAergic neurons from the ganglionic eminence. This repulsive effect is blocked by anti-DCC antibodies, indicating the involvement of DCC in this repulsion, possibly by complexing with the Unc5 receptor. By contrast, Marin et al. (2003) reported that netrin-1 does not contribute to the tangential cortical migration of GABAergic interneurons. Mice with genetic deletion of netrin-1, as well as triple-knockouts for Slit1 and Slit2 (expressed in the subpallium) in addition to netrin-1, exhibit a normal distribution of cortical interneurons at E18 (Marin et al., 2003). Nevertheless, Stanco et al. (2009) found that netrin-1 in the marginal zone and intermediate zone guides tangential migration of ganglionic eminence-derived interneurons, which is mediated by α3β1-integrin (Figure 2A). *In vivo* analysis of interneuron-specific α3β1-integrin- and netrin-1-deficient mice revealed abnormal interneuron migration along the top of the developing cortical plate, disrupting the distribution of interneurons throughout the cerebral cortex including the hippocampus. The interactions between the C terminus of netrin-1 and α6β4 and α3β1 integrins are also known to contribute to cell adhesion as well as to the migration of non-neuronal cells, such as pancreatic epithelial cells and mesenchymal stem cells (Yebra et al., 2003; Son et al., 2013; Lee et al., 2016). The binding of netrin-4 to α6β1 integrin, which makes a ternary complex with laminin γ1, promotes neurogenesis and migration in the rostral migratory stream (Staquicini et al., 2009). Furthermore, other combinations of netrin-4/integrin interactions are reported in non-neuronal systems. Namely, α2β1, α3β1, α6β1, and b4 integrins bind to netrin-4 on endothelial cells, epithelial cells, and glioblastomas (Larrieu-Lahargue et al., 2011; Yebra et al., 2011; Hu et al., 2012). Although these netrin–integrin interactions have not been extensively analyzed in neuronal cells, they might contribute to cortical migration.

Netrin-4 is known to influence the maturation of cortical neurons and is highly expressed in pyramidal cells of the neocortex and hippocampus, and Purkinje cells of the cerebellum (Zhang et al., 2004). Netrin-4 can bind to DCC and Unc5 receptors via its N-terminal domain, although an unidentified receptor can bind to its C-terminal domain. In layer 4 neurons in the visual cortex and somatosensory area, both netrin-4 and its receptor Unc5D are expressed (Figures 2A,C). As the Unc5D receptor has an intracellular death domain that triggers apoptosis without ligand binding as a dependent receptor, netrin-4 seems to serve to inhibit apoptotic cell death (Takemoto et al., 2011). Furthermore, netrin-4/Unc5B signaling regulates the branching of thalamocortical neuron axons in the somatosensory and visual cortices in an activity-dependent manner (Hayano et al., 2014). The contribution of netrin-4 to cortical migration has not yet been analyzed. However, according to Allen Developing Mouse Brain Atlas^1^, netrin-4 expression does not occur at E15.5 and E18.5 in the cerebral cortex. Therefore, instead of functioning in cortical cell migration, netrin-4 may play a role in cell survival and maturation.

**TABLE 1:** List of references to publications describing the expression patterns of netrin family proteins and their interacting proteins in the cerebral cortex depicted in Figure 2.

Name	Age	Method	References
*Netrin-1*	E13.5	LacZ	Stanco et al. (2009)
Netrin-1	E13.5	IHC	Stanco et al. (2009)
*Netrin-1*	E14–15	ISH	Yamagishi et al. (2011); Miyoshi and Fishell (2012)
*Netrin-4*	E15.5	ISH	Yamagishi et al. (2011)
*Netrin-4*	P6	ISH	Takemoto et al. (2011)
*Netrin-G1*	E16.5	ISH	Nakashiba et al. (2002)
*Netrin-G2*	E18, P0	ISH	Allen Developmental Mouse Brain Atlas; Nakashiba et al. (2002)
*DCC*	E14.5	ISH	Miyoshi and Fishell (2012)
Neogenin	E14.5	IHC	Fitzgerald et al. (2006)
*DsCAM*	E14.5	ISH	Miyoshi and Fishell (2012)
Draxin	E14.5	IHC	Shinmyo et al. (2015)
*Draxin*	E17.5	LacZ	Shinmyo et al. (2015)
*Unc5a*	E14.5	ISH	Miyoshi and Fishell (2012)
*Unc5b*	E14.5	ISH	Miyoshi and Fishell (2012)
*Unc5c*	E14.5	ISH	Srivatsa et al. (2014)
*Unc5c*	P0	ISH	Srivatsa et al. (2014)
*Unc5d*	E14.5–16.5	ISH	Takemoto et al. (2011); Yamagishi et al. (2011); Miyoshi and Fishell (2012)
*Unc5d*	P6	ISH	Takemoto et al. (2011); Yamagishi et al. (2011)
*FLRT1*	E15.5	LacZ	Del Toro et al. (2017)
FLRT1	E15.5	IHC	Del Toro et al. (2017)
*FLRT2*	E15.5	ISH, LacZ	Yamagishi et al. (2011); Del Toro et al. (2017)
*FLRT2*	P0	ISH	Yamagishi et al. (2011)
*FLRT3*	E15.5	LacZ	Del Toro et al. (2017)
FLRT3	E15.5	IHC	Del Toro et al. (2017)

GPI-anchored netrin G1 and G2 show distinctive expression patterns. During the development of the cerebral cortex, netrin G1 is expressed in the marginal zone and subplate, whereas netrin G2 is expressed throughout the cortex (Figure 2A) (Nakashiba et al., 2002). Knockdown of either netrin G1 or G2 at E14.5 by IUE impairs radial migration at postnatal day 1 and 7 (Heimer et al., 2020). Netrin-G1 knockdown results in a major migration defect, with only ∼50% of cells entering the cortical plate at postnatal day 1 and ∼60% of transfected cells managing to migrate to layer 2/3. Netrin-G2 knockdown shows a similar migration deficit. Only 55% and 40% of transfected cells reached to the cortical plate at P1 and layer2/3 at P7, respectively (Heimer et al., 2020).

To the best of our knowledge, there is no other publication showing the function of netrin family proteins in either radial or tangential migration of cortical neurons. In 2015, we reported another member of netrin protein family, netrin-5, which lacks the N-terminal laminin VI domain (Yamagishi et al., 2015). It is not well characterized because netrin-5 mutant mice develop normally without any obvious phenotypes (Garrett et al., 2016). Garrett et al. (2016) also showed high expression of netrin-5 in the boundary cap cells (BCCs) in the spinal cord, which prevents migration into the ventral root. When netrin-5 is absent in BCCs, subsets of motor neurons migrate to the peripheral nervous system. Interestingly, this phenocopies DCC^–/–^ mice, suggesting that the interaction between netrin-5 and DCC induces a repulsive signal, possibly together with the Unc5 receptor. Biochemical screens revealed that DSCAM is another receptor for netrin-5 (Visser et al., 2015); however, the function of this interaction is not known, as *in vitro* growth cone collapse and turning assays have not been performed. Recently, we reported that netrin-5 is involved in organizing the rostral migratory stream in the adult mouse brain (Ikegaya et al., 2020). However, the contribution of netrin-5 to cortical development remains to be determined.

## Roles of DCC in the Migration of Cortical Neurons

Deleted in colorectal cancer (DCC) regulates the radial migration of cortical neurons. Zhang et al. (2018) reported that DCC interacts with Dab1, an intracellular transducer of Reelin signaling, by binding to ApoER2 and VLDLR in multipolar migrating neurons. Netrin-1 induces Dab1 phosphorylation, and knockdown or truncation of the C-terminal P3 domain of DCC impairs the multipolar-to-bipolar transition of neurons, dramatically delaying their migration. These results indicate that Dab1 mediates netrin-1/DCC signaling. Myosin-10 (Myo10), a non-traditional myosin family member, interacts with DCC for radial migration (Ju et al., 2014). Full-length Myo10 is expressed in the VZ/SVZ, and headless Myo10 is expressed in the intermediate zone as well as in the VZ/SVZ. Knockdown of full-length Myo10 results in abnormally oriented bipolar neurons, whereas knockdown of the headless isoform impairs the multipolar–bipolar transition. Interestingly, overexpression of DCC rescues the full-length Myo10 knockdown phenotype but not the headless Myo10 knockdown phenotype, indicating that DCC is involved in full length Myo10-regulated migration, but not in headless Myo10-controlled morphological transition. Although the downstream signaling of DCC in migrating neurons is not fully understood, *in vitro* analyses of non-neuronal cells suggest that FAK, Nck1, Rac, cdc42, and RhoA may be involved in the netrin-DCC signaling pathway (Li et al., 2002a,b; Shekarabi and Kennedy, 2002).

## Neogenin/RGM

Neogenin, which is expressed throughout the telencephalon, including in dividing neuroepithelial cells, at E12.5 (Fitzgerald et al., 2006), is involved in the migration of both excitatory and inhibitory neurons. At E14.5, neogenin is expressed on nestin- and GLAST-positive radial glia and within the VZ, SVZ, and intermediate zone of the cortex (Figure 2B). The expression of neogenin on neurons migrating through the intermediate zone is turned off once they reach the cortical plate, where they begin expressing DCC. Thus, neogenin expression is limited to the immature stage of excitatory neurons, but is also expressed by newborn cortical interneurons and the maturing calbindin- and parvalbumin-positive subpopulations (O’Leary et al., 2013). The ligand of neogenin is RGMa, which provides a repulsive cue for newborn interneurons migrating away from the VZ and medial ganglionic eminence. Interestingly, this repulsion is suppressed by netrin-1, suggesting that RGMa and netrin-1 compete for binding to neogenin to control migration. The expression of neogenin is regulated by Rb, a tumor suppressor (Andrusiak et al., 2011). In Rb mutant mice in which expression is driven by Foxg1-cre recombinase, neogenin is strongly upregulated in the telencephalon. This results in augmented interneuron adhesion and a defective migratory response to netrin-1 *in vitro* (Andrusiak et al., 2011). *In vivo*, overexpression of neogenin impairs migration of neuroblasts from the SVZ and medial ganglionic eminence.

Radial migration is regulated in a RGMa/neogenin dependent manner. RGMa is expressed in the cortical plate and VZ. Knockdown of neogenin in migrating neurons results in their abnormal distribution to the areas where RGMa is expressed (van Erp et al., 2015). A similar phenotype was observed with knockdown of Lrig2, a negative regulator of the proteolytic cleavage of neogenin by ADAM17, as this results in RGMa insensitivity. These results indicate that RGMa-neogenin-Lrig2 signaling propels migrating neurons out of the VZ/SVZ and prevents their premature entry into the cortical plate (van Erp et al., 2015). Interestingly, Unc5B can interact with neogenin as a coreceptor for RGMa (Hata et al., 2009), such that knockdown of the Unc5 receptor eliminates the repulsion mediated by RGMa, including growth cone collapse in cortical neurons *in vitro*. However, whether the Unc5 family protein is involved in the RGMa-mediated repulsion in tangential and radial migration *in vivo* remains unknown.

## Down Syndrome Cell Adhesion Molecule (DSCAM)

Down syndrome cell adhesion molecule, another factor regulating radial migration, is a large (>200 kDa) neural cell adhesion molecule that consists of 10 Ig C2-type domains, six FN type-III domains, a transmembrane domain, and a C-terminal intracellular domain. DSCAM and DSCAML1, a splice variant, are widely expressed in all layers of the cerebral cortex. More precisely, DSCAM is highly expressed in layer V, whereas DSCAML1 is more prominent in the superficial layer and layer V. Knockdown of either DSCAM or DSCAML1 impairs the radial migration of upper layer neurons at P0. The DSCAMs deficient neurons remained trapped in the deep layers and intermediate zone, which was rescued by overexpression of full length DSCAM. At P7, a large number of shDSCAM-transfected neurons failed to migrate to layers II–III, whereas most shDSCAML1-transfected neurons did (Zhang et al., 2015). These knockdowns disrupt the callosal projections of cortical neurons to the contralateral hemisphere, and increase the dendritic branching in cultured cortical neurons. However, it is not clear whether these phenotypes involve netrin family proteins.

## Draxin

Draxin (dorsal repulsive axon guidance protein) binds to various netrin-related proteins. Islam et al. (2009) characterized draxin as a repulsive cue regulating midline crossings of axons in the corpus callosum, hippocampal commissure, anterior commissure, and commissure neurons of the spinal cord, regulation that is phenocopied in netrin-1^–/–^ and DCC^–/–^ models. Indeed, draxin directly interacts with netrin-1 and DCC, as well as with DSCAM and Unc5a-c (Ahmed et al., 2011; Gao et al., 2015; Meli et al., 2015; Liu et al., 2018). In draxin knockout mice, not only midline-crossing commissural axons but also thalamocortical and corticofugal projections are severely affected (Shinmyo et al., 2015). Interestingly, draxin promotes the growth of thalamic neuron axons *in vitro*, which is abolished by DCC deficiency, indicating that draxin acts as an attractant, similarly to netrin-1. Although it is highly expressed in the developing cortex, neither radial migration nor tangential migration of interneurons is affected by draxin deficiency (Figure 2C) (Shinmyo et al., 2015). These results suggest that draxin is not involved in neuronal migration, but rather is specifically involved in axon guidance, unlike netrin-1 and DCC. It is also possible that another molecule compensates for the absence of draxin to ensure proper cortical organization of neuronal migration.

## Unc5/FLRT

Among four members of Unc5 protein family, Unc5b regulates interneuron migration and Unc5d regulates radial migration. During tangential migration to the cortex, GABAergic interneurons express transcription factor Sip1, also known as ZEB2 or Zfhx1b, which regulates Unc5b expression (van den Berghe et al., 2013). In Sip1 mutant mice, interneurons exhibit a migration defect, and Unc5b and netrin-1 are highly upregulated. Overexpression of Unc5b, but not netrin-1, contributes to the migration defect. Furthermore, Unc5b knockdown rescues the aberrant migration in Sip1 mutants, indicating that downregulation of Unc5b by Sip1 is necessary for normal interneuron migration (van den Berghe et al., 2013).

Unc5d is the most-characterized molecule among four Unc5 family proteins involved in radial migration. A portion of the *Unc5d* gene was first characterized as an *in situ* probe, *Svet1*, a specific marker of the embryonic SVZ and the upper layers of the mature cortex (Figure 2C) (Tarabykin et al., 2001). *Svet1* cDNA consists of 3,934 bp without an open reading frame and was later identified as part of a 324 kb intron between exon 1 and exon 2 of *Unc5d* (Sasaki et al., 2008). *Svet1/Unc5* is expressed in multipolar neurons in the SVZ, which migrate to the upper layers. Interestingly, when the neurons migrate through deep layers, where a high-affinity repulsive ligand to Unc5d, FLRT2, is expressed, Unc5d is temporarily shut down by the suppression of nuclear RNA splicing. Upon arrival to the upper layer, Unc5D is re-expressed (Yamagishi et al., 2011). Overexpression of Unc5d delays radial migration, whereas knockout of *Unc5d* results in broader distribution of Tbr2^+^ intermediate progenitor cells, typically confined to the SVZ, toward the cortical plate (Yamagishi et al., 2011; Seiradake et al., 2014). Such dynamic expression of Unc5d is highly regulated by transcription factor FoxG1 (Miyoshi and Fishell, 2012). FoxG1 gain-of-function cells fail to express Unc5d and show a migration defect, which is rescued by Unc5d overexpression, whereas a loss of FoxG1 function arrests cells in an early multipolar phase. Upregulation of FoxG1 is required to exit the multipolar cell phase and to enter the cortical plate. Furthermore, *in situ* pattern analysis revealed that *Unc5d* and *Dcc* were among the genes with the highest expression induced by *Eomes* (Tbr2) (Cameron et al., 2012).

The zinc-finger transcriptional repressor, RP58, controls the multipolar-to-bipolar transition by suppressing the neurogenin2–Rnd2 pathway (Heng et al., 2008; Ohtaka-Maruyama et al., 2012, 2013). RP58 forms a transcriptional complex with FoxG1, and chromatin immunoprecipitation sequencing revealed associations with *Neurog2*, *NeuroD1*, *Rnd2*, and *Unc5D* (Cargnin et al., 2018). Another transcription factor, PRDM8, regulates multipolar-to-bipolar transition by modulating Unc5d levels (Inoue et al., 2014). Although the expression patterns of PRDM8 and Unc5d partially overlap, overexpression of PRDM8 inhibits Unc5d expression and *vice versa*.

FLRT family proteins are involved in the radial migration and gyrus formation of the cerebral cortex. High-affinity binding of FLRT2 to Unc5D (*K*_*d*_ = 0.31 μM) induces a repulsive signal and controls radial migration (Yamagishi et al., 2011; Seiradake et al., 2014). FLRT proteins also bind Latrophilin3 (Lphn3), which is involved in cell adhesion and synaptogenesis (*K*_*d*_ = 40 nM) (O’Sullivan et al., 2012; Jackson et al., 2015). In addition, FLRT2/Unc5D/Lphn3 forms a ternary complex in a stoichiometry of 1:1:2, which further dimerizes to make a larger supercomplex at 2:2:4 (Lu et al., 2015; Jackson et al., 2016). As FLRT and Lphn also form a ternary complex with teneurin (Sando et al., 2019; Del Toro et al., 2020), it would be interesting to know whether they form a large tetra-complex with Unc5. The multiple FLRT bindings with repulsive/adhesive functions play important roles in radial migration, tangential distribution, and synapse formation. Indeed, FLRT1/3 double-knockout mice show ectopic cortical gyrus formation (Del Toro et al., 2017). The expression pattern of FLRTs is summarized in Figure 2D. The functions of FLRTs in cortical migration and gyrus formation have been the subject of a previous review article (Peregrina and del Toro, 2020).

## Human Diseases

Recent genetic analyses have revealed that mutations in the above mentioned guidance molecules are involved in congenital disorders. Mutations in genes encoding netrin-1 (*NTN1*) and DCC (*DCC*) in human result in abnormal targeting of corticospinal tracts and congenital mirror movements, a disorder characterized by involuntary movements of one hand that mirror the intentional movements of the opposite hand. Three mutations in exon 7 of *NTN1*, I518del, C601R, and C601S, encoding the C-terminal NTR domain, were identified in two unrelated families and one sporadic case. In the patients, only a portion of the corticospinal tracts crossed at the medulla, resulting in uncrossed aberrant corticospinal tract projections to ipsilateral motor neurons, as well as contralateral projections (Depienne et al., 2011; Méneret et al., 2017). Since the netrin-1 mutation causes the abnormal projection of cortical spinal tract, it is plausible that netrin-1 is not relevant to cortical migration. On the other hand, netrin-1 is expressed in neurons and oligodendrocytes in the spinal cord and regulates radial and tangential neuronal migration (Manitt et al., 2001; Junge et al., 2016). Therefore, it is possible that the abnormal positioning of neurons indirectly affects the distribution of the corticospinal tract. Also, short-range netrin-1 effects might be associated with the maintenance of appropriate neuronal and axon–oligodendroglial interactions and/or maintenance of synaptic interactions and plasticity in the mature nervous system.

Netrin-1 is expressed in the marginal zone and intermediate zone in the mid-gestation stage of mouse, but its expression disappears thereafter (Figure 2) (Livesey and Hunt, 1997). However, limited netrin-1 expression is observed in the medial prefrontal cortex, which dopaminergic neurons innervate from the midbrain (Manitt et al., 2011). Notably, several psychiatric disorders, including schizophrenia, depression, and drug abuse, are associated with altered organization and function of mPFC circuitry (Tan et al., 2007; Davey et al., 2008; Feil et al., 2010). Indeed, a genome-wide methylation study of twins revealed that *Netrin-1* had an altered methylation status in patients with depression (Roberson-Nay et al., 2020). Another study showed an association between a SNP in Netrin-1 (rs8081460) and neuroticism (Smith et al., 2016).

Recent genome-wide association studies have revealed that a growing number of *DCC* mutations are associated with psychiatric disorders, such as mood instability, neuroticism, schizophrenia, and depression (Ward et al., 2017; Kibinge et al., 2020; Li et al., 2020; Torres-Berrío et al., 2020; Vosberg et al., 2020). In patients with depression, the expression of DCC is abnormally high in the dorsolateral prefrontal cortex, which connects to the thalamus, caudate nucleus, hippocampus, orbitofrontal cortex, and other cortical areas (Li et al., 2020). Furthermore, *DCC* mRNA levels in prefrontal cortex were ∼40% higher in patients who committed suicide (Manitt et al., 2013; Torres-Berrío et al., 2017). A murine model with depression-like symptoms induced by chronic social defeat stress also exhibits higher levels of DCC in the prefrontal cortex (Torres-Berrío et al., 2017). Individuals with *DCC* haploinsufficiency exhibit reduced striatal volumes and impaired connectivity between the substantia nigra and the ventral tegmental area and ventromedial prefrontal cortex, resulting in lower novelty-seeking scores (Vosberg et al., 2018).

Although there is no direct evidence that aberrant cortical migration caused by DCC mutations is involved in the above mentioned phenotypes, a cortical migration defect is known to cause psychiatric disorders, such as schizophrenia (Muraki and Tanigaki, 2015) and decreased novelty recognition (Hamada et al., 2017). Indeed, miRNA knockdown of the psychiatric illness risk gene DISC1 affects the tangential migration of interneurons (Steinecke et al., 2012). Therefore, DCC-associated psychiatric disorders may be caused by abnormal cortical migration. In patients with schizophrenia, abnormal cortical layers or cell distributions have been reported. Iritani et al. (1999) reported that calbindin-D28K positive cells are distributed abnormally in the prefrontal cortex (Brodmann area 9). Cajal–Retzius cells, which produce reelin signals, are more numerous in the lower third of layer I in schizophrenia patients (Kalus et al., 1997). Again, since netrin-1 is expressed in layer I and IZ, and involved in interneuron migration during the gestation stage (Figure 2), these abnormal distributions may explain the correlation between psychiatric disorders associated with *Netrin-1* and *DCC* mutations (Vosberg et al., 2020).

Single nucleotide polymorphisms or abnormal expression of *NTNG1* and *NTNG2* are related to psychiatric disorders such as autism, Rett syndrome, schizophrenia, and bipolar disorder in human and murine models (Fukasawa et al., 2004; Aoki-Suzuki et al., 2005; Chuang et al., 2015; Huang and Hsueh, 2015; Heimer et al., 2020). The levels of *NTNG1* mRNA, especially isoform G1c, and *NTNG2* are decreased in patients with bipolar disorder (Eastwood and Harrison, 2008). However, the same group later reported elevations of netrin G1d and G1f isoforms and of netrin G2 in patients with bipolar disorder, indicating that an alteration of netrin G1 levels is critical for susceptibility to the disease (Eastwood and Harrison, 2010). Abnormally high expression of netrin G2 was observed in the temporal lobes of patients with intractable epilepsy, as well as in a rat model involving the use of pilocarpine (Pan et al., 2010). Mutations in the *NTNG1* gene are linked to Rett syndrome with epileptic seizures of early onset (Archer et al., 2006; Nectoux et al., 2007), and a more recent study revealed that netrin G2 dysfunction is associated with a Rett-like phenotype with areflexia (Heimer et al., 2020).

A large-scale single nucleotide polymorphism analysis of chromosome 4 revealed that *UNC5C* is one of the susceptibility genes for schizophrenia, bipolar disorder, and depression (Tang et al., 2019). Moreover, several studies revealed that single nucleotide polymorphism of *UNC5C* is relevant to Alzheimer’s disease (Sun et al., 2016; Cukier et al., 2017; Yang et al., 2017). The level of Unc5c was decreased in the dorsolateral prefrontal cortices of patients with Alzheimer’s disease-related cerebral amyloid angiopathy (Yang et al., 2017). In addition, a rare mutation, T835M, of *UNC5C* was identified from parametric linkage analysis of late-onset Alzheimer’s disease (Wetzel-Smith et al., 2014). Using a mouse model, the authors revealed that T835M-expressing neurons were more vulnerable to Aβ-induced neurotoxicity than controls. However, whether there is a link between cortical migration and the onset of the these diseases remains an open question.

## Future Directions

The last two decades of research have seen an expansion of the involvement of netrins in axon guidance to multiple physiologic and pathophysiologic functions such as synapse formation/plasticity, learning/memory, neuronal migration, and psychiatric disorders. However, little is known about how these multiple events are spatially and temporally coordinated in axons and dendrites for proper wiring of neuronal networks after radial and tangential migration, which is orchestrated by the guidance molecules and other extracellular cues. “Multi-omic” studies of netrins and other related genes, including transcriptomic, proteomic, and metabolomics approaches, could help identify other key targets impacting neuronal migration and its downstream events. In addition, spatially and temporally confined deletion of the guidance genes could shed light on crucial mechanisms involved in the dynamic regulation of cortical migration.

Interestingly, many mutations in netrins and their related molecules cause psychiatric disorders, such as schizophrenia, bipolar disorder, mood instability, neuroticism, depression, autism, and Rett syndrome, as mentioned above. However, the detailed molecular mechanisms via which mutations in these genes cause these diseases remain unclear. Although netrins and their receptors are known risk factors for these diseases, other extracellular molecules such as laminins and proteoglycans may also interact with netrin signaling cascades and lead to the onset of these diseases. Further investigations are needed to fully understand whether or not abnormalities in cortical migration contribute to psychiatric disorders. The results of such investigations combined with the identification of netrin signaling targets may open new avenues for understanding and treating neurological disorders.

## Author Contributions

All authors listed have made a substantial, direct and intellectual contribution to the work, and approved it for publication.

## Conflict of Interest

The authors declare that the research was conducted in the absence of any commercial or financial relationships that could be construed as a potential conflict of interest.

## Footnotes

1 http://developingmouse.brain-map.org
